# Vaccination coverage and serological follow-up in children with rheumatologic diseases: a retrospective single-centre study

**DOI:** 10.1186/s12969-026-01240-5

**Published:** 2026-06-23

**Authors:** Ilias Grafas, Laure F. Pittet, Manel Mejbri, Geraldine Blanchard-Rohner

**Affiliations:** 1 Faculty of Medicine, University of Geneva, Geneva, Switzerland; 2 Research Platform of Pediatrics, Gynecology, and Obstetrics, Department of Pediatrics, Gynecology and Obstetrics, Geneva University, Geneva, Switzerland; 3 Unit of Immunology, Vaccinology, and Rheumatology, Division of General Pediatrics, Department of Pediatrics, Gynecology and Obstetrics, Geneva University Hospitals and University of Geneva, Geneva, Switzerland

**Keywords:** Rheumatologic diseases, Vaccination coverage, Vaccine seroprotection, Measles, Varicella, Hepatitis B, Haemophilus influenzae type b, Pneumococcus, Pediatric rheumatology, Immunosuppression

## Abstract

**Background:**

Children with rheumatologic diseases often require immunosuppressive therapies, which can compromise their immune response and increase susceptibility to infections. Despite the heightened risk, vaccination coverage in this population is frequently suboptimal, largely due to concerns over vaccine safety, especially live attenuated vaccines, and the absence of clear, universally adopted guidelines.

**Objective:**

This retrospective study aims to evaluate the vaccination status and serological immunity of pediatric patients with rheumatologic diseases at the time of diagnosis, prior to initiation of immunosuppressive therapy.

**Methods:**

We conducted a retrospective review of medical records from children under 16 years of age diagnosed with a rheumatologic disease at Geneva University Hospitals (HUG) between 2005 and 2023. Demographic data, clinical diagnosis, vaccination history, and available vaccine-specific serologies were collected and analysed.

**Results:**

Seventy-four patients were included, with a median age at diagnosis of 6 years. At the time of diagnosis, vaccination coverage was highest for *Haemophilus influenzae* type b (Hib; 93%), followed by diphtheria and tetanus (91%), measles-mumps-rubella (MMR; 80%), pertussis and poliomyelitis (77–78%), pneumococcus (77%), varicella-zoster virus (VZV; 72%), and hepatitis B virus (HBV; 64%). Baseline serological testing was available in only half of the patients (38/74, 51%). Among those tested, seroprotection was highest for Hib (100%), followed by tetanus (91%), measles (89%), diphtheria (88%), hepatitis B (58%), varicella (47%), and pneumococcus (42%). Longitudinal follow-up revealed limited and inconsistent serological monitoring, with only a minority of patients undergoing repeat testing, and very few receiving booster vaccinations, despite the generally stable protection levels observed over time.

**Conclusions:**

Vaccination coverage in children with rheumatologic diseases remains suboptimal, particularly for varicella, hepatitis B, and pneumococcus. These findings underscore the critical importance of optimizing vaccination coverage at diagnosis and establishing systematic serologic surveillance protocols in this high-risk population, rather than relying solely on vaccine history.

**Supplementary Information:**

The online version contains supplementary material available at https://doi.org/10.1186/s12969-026-01240-5.

## Introduction

Pediatric rheumatologic diseases encompass a heterogeneous group of conditions affecting children, including both autoimmune inflammatory rheumatic diseases (AIRDs)—such as Juvenile Idiopathic Arthritis (JIA), the most prevalent, Systemic Lupus Erythematosus (SLE), Juvenile Dermatomyositis (JDM), and various types of vasculitis—and autoinflammatory diseases, such as Familial Mediterranean Fever (FMF), which are driven by innate immune dysregulation rather than adaptive immune responses and are typically managed with colchicine rather than conventional immunosuppressants. These conditions primarily affect the joints, connective tissue, skin, muscles, and internal organs. They are typically chronic and systemic, characterized by periods of remission and relapse, often requiring long-term immunomodulatory or immunosuppressive therapy to control inflammation and prevent irreversible tissue damage [1]. The impact of these conditions extends beyond physical health, influencing psychosocial development, schooling, and family dynamics.

The pathophysiology of pediatric rheumatic diseases commonly involves a dysregulated immune response in which the body’s immune system erroneously targets its own cells and tissues. This inappropriate immune activation leads to chronic inflammation and tissue destruction, necessitating the use of immunosuppressive medications such as corticosteroids, methotrexate, biologics (e.g., TNF-alpha inhibitors), and other disease-modifying antirheumatic drugs (DMARDs) [2]. While these therapies are critical for disease control and improved long-term outcomes, they may impair immune responses, placing children at increased risk for both common infections (e.g., respiratory tract infections) and more severe, potentially life-threatening infections [3, 4].

In this context, vaccination becomes a vital preventive measure. Immunization not only reduces the frequency of infections but also lowers the risk of hospitalization and complications, particularly in immunosuppressed patients [5, 6]. Nevertheless, vaccination strategies in this population remain complex and often inadequate. Despite strong evidence supporting the safety and efficacy of non-live vaccines in immunosuppressed children, vaccination coverage remains suboptimal due to several barriers, including parental hesitancy, clinical uncertainty, inconsistent adherence to follow-up, and lack of universal guidelines [7–11]. The use of live attenuated vaccines, such as those for measles, mumps, and rubella (MMR) or varicella, is more controversial due to the potential risk of vaccine-derived infection in immunocompromised individuals [12, 13].

The European League Against Rheumatism/Paediatric Rheumatology European Society (EULAR/PRES) 2021 guidelines provide updated recommendations on the safe administration of vaccines in children with AIRDs [10]. These include a preference for vaccinating prior to the initiation of immunosuppressive therapy, routine use of inactivated vaccines regardless of treatment phase, and cautious consideration of live attenuated vaccines depending on the degree of immunosuppression [10]. Importantly, EULAR/PRES guidelines do not recommend delaying immunosuppressive therapy in order to complete vaccination, as the window of opportunity to treat—the treat-to-target principle—takes precedence; the time of diagnosis should rather be used to update vaccinations concurrently or immediately prior to starting therapy whenever feasible. However, gaps remain in clinical implementation. Factors such as age at diagnosis, timing of therapy initiation, and variable national immunization schedules contribute to incomplete vaccination coverage [14, 15]. As a result, some children may enter long-term treatment already under-immunized or fall behind on routine immunizations during the course of their disease.

In this context, we conducted a retrospective study to assess the vaccination status and serological immunity of children diagnosed with rheumatologic diseases. By analyzing immunization records and vaccine serology data, the study seeks to provide insight into the level of protection in this high-risk population and identify opportunities for improving vaccination practices in pediatric rheumatology.

## Method

### Study setting and population

This retrospective observational study was conducted at the Geneva University Hospitals (HUG), Switzerland. Patients had to be followed at HUG, diagnosed with a rheumatologic disease between 2005 and 2023, and under 16 years old at the time of diagnosis. Patients had to have an available vaccination record and eligible for an immunosuppressive treatment during the study period. The study was approved by the local ethics committee under reference CCER 2020–01537.

### Data collection

Clinical data was collected from the electronic patient records and entered into a Research Electronic Data Capture (REDCap) database [16]. Given the lack of vaccination data in the HUG electronic database, parents and pediatricians were contacted to retrieve as much vaccination data as possible. Vaccination data, along with vaccine serology and demographic information were collected.

### Definition of vaccine status and seroprotection

Vaccine status was classified as “up-to-date” or “not-up-to-date” depending on the Swiss vaccination guidelines for each vaccine according to the patient’s age (see supplementary Table S1) [17]. This approach was selected as it most closely resembles clinical practice, where vaccinations are updated at diagnosis to the current schedule. Patients not yet eligible for a given vaccine by age were therefore considered up-to-date.

Vaccination and history of natural infections were obtained from the patient’s vaccination record, which are usually recorded to the hospital electronic patient record.

Vaccine serologies were performed as part of routine follow-up and analyzed at HUG for tetanus, diphtheria, *Haemophilus influenzae* type B (Hib), pneumococcus, measles, varicella, and hepatitis B.

Vaccine seroprotection was defined according to laboratory cut-offs used at the HUG and are as follows: varicella (150 IU/L), measles (150 IU/L), diphtheria (100 IU/L), tetanus (100 IU/L), Hib (0.15 mg/L), and hepatitis B (10 IU/L) [18].

Regarding pneumococcal immunity, patients were considered “seroprotected” if they had serotype-specific immunoglobulins G (IgG) levels > 0.50 mg/L for 2 over 3 representative serotypes (14, 19F, and 23F) included in the 7- and 13-valent conjugated vaccine (Prevenar®/-13®), measured with an enzyme-linked immunosorbent assay [19]. After 2019, seroprotection was defined by having serotype-specific levels > 0.50 mg/L to at least 4 of the 7 tested serotypes (4, 6B, 9 V, 14, 18C, 19F, and 23F, all included in the 13-valent conjugated vaccine), measured with multiplex binding assay [20, 21].

### Statistical analysis

Categorical data are presented as frequencies and percentages. Continuous data are reported as geometric mean concentrations (GMC). Due to the descriptive nature of our study, no sample size calculation was performed; all patients at our center who met the inclusion criteria were included.

## Results

### Demographics

**Fig. 1:**
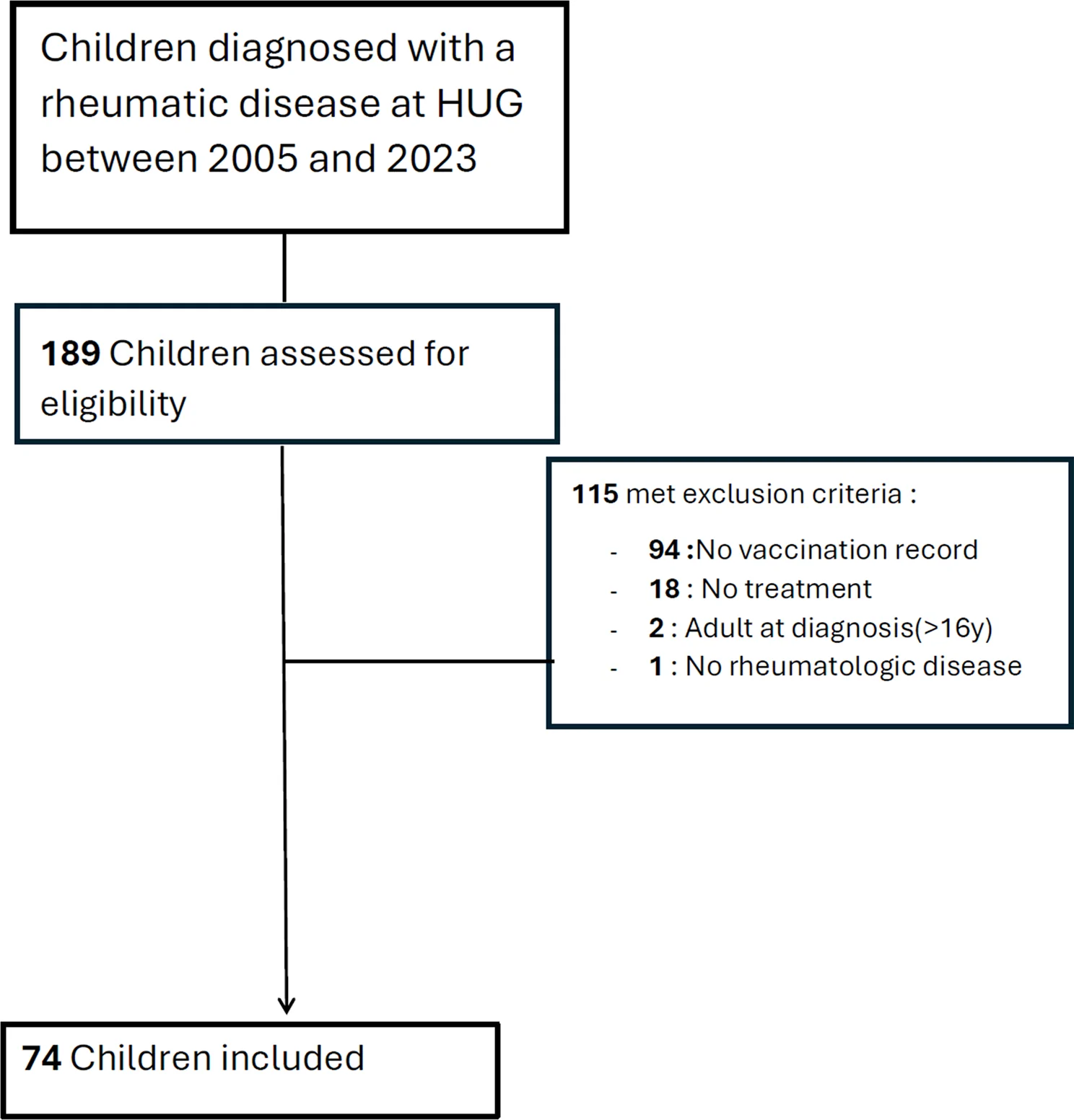
Flow chart

Of the 189 patients screened, 94 were excluded due to lack of vaccination data available, 18 did not receive immunosuppressive therapy, 2 were already adults at the time of diagnosis, and 1 patient was excluded due to a final diagnosis of a non-rheumatologic condition (IBD). As a result, 74 patients met the inclusion criteria for this study, as illustrated in Fig. 1.

Among the included patients, the median age at diagnosis was 6 years. The cohort was predominantly female (73%, *n* = 54) and most patients were of European origin (80%, *n* = 59). The most frequently diagnosed conditions were juvenile idiopathic arthritis (JIA) (51%, *n* = 38), uveitis (12%, *n* = 9), familial Mediterranean fever (10%, *n* = 8), and SLE (9%, *n* = 7). Uveitis was listed as a separate diagnostic category only when it represented the primary indication for systemic treatment, in the absence of an underlying rheumatic disease; JIA-associated uveitis was classified under the JIA category. Regarding treatment, the most commonly prescribed medications were methotrexate (31%, *n* = 23), followed by etanercept (Enbrel) and adalimumab (Humira), each used in 14% (*n* = 10) of cases. Further details on diagnoses and treatments are provided in Table 1. Of note, 9 patients were treated with colchicine: 8 had a diagnosis of FMF, while the ninth had a diagnosis of TRAPS (TNF receptor-associated periodic syndrome), another autoinflammatory disease for which colchicine is sometimes prescribed, categorized under ‘Other’ in Table 1.

**Table 1:** Demographic characteristics of the study population

Characteristic	Value n, (%)
**Age at Diagnosis in Years, Median [IQR]**	6 [6]
**Sex, n(%)**	
Female	54 (73%)
**Nationality, n(%)**	
Europe	59 (80%)
Asia	8 (11%)
Africa	4 (5%)
South America	3 (4%)
**Diagnosed Diseases**, n(%)	
Juvenile idiopathic arthritis	38 (51%)
Uveitis	9 (12%)
Familial mediterranean fever	8 (11%)
Systemic lupus erythematosus	7 (9%)
Juvenile dermatomyositis	2 (3%)
Other	10 (14%)
**Treatments**, n(%)	
Méthotrexate ± prednisone	23 (31%)
Étanercept ± méthotrexate ± prednisone	10 (14%)
Adalimumab ± méthotrexate ± prednisone ± azathioprine	10 (14%)
Colchicine	9 (12%)
Tocilizumab	6 (8%)
Mycophénolate mofétil ± prednisone	6 (8%)
Canakinumab	2 (3%)
Prednisone	2 (3%)
Tacrolimus	1 (1%)
Rituximab	1 (1%)
Secukinumab	1 (1%)
Sirolimus	1 (1%)
Abatacept	1 (1%)
Anakinra	1 (1%)

*include Sjögren’s syndrome, microscopic polyangiitis, Interferonopathy, granulomatosis with polyangiitis, TNF receptor-associated periodic syndrome (TRAPS), undetermined myositis, chronic osteomyelitis, scleroderma, unexplained fever syndrome

One patient treated with colchicine carried a diagnosis of TNF receptor-associated periodic syndrome (TRAPS), included in the “Other” diagnostic category

Uveitis was listed as a separate diagnostic category only when it represented the primary indication for systemic treatment, in the absence of an underlying rheumatic disease; JIA-associated uveitis was classified under the JIA category (*n* = 7)

### Vaccination coverage

At the time of diagnosis, vaccination coverage was highest for *Haemophilus influenzae* type b (Hib), documented in 63 patients (93% of children > 5 years), followed by diphtheria and tetanus toxoids, with 67 patients (91%) up to date, both typically administered as part of the DTaP-IPV-Hib-HepB combination vaccine. For measles, mumps, and rubella, administered together as the MMR vaccine, 59 patients (80%) were up to date at diagnosis. Regarding pertussis and poliovirus, both antigens included in the hexavalent combination vaccine, 57 and 58 patients (77 and 78% respectively) had received age-appropriate doses. The slight discrepancy between polio and pertussis coverage rates can be explained by the choice of booster vaccine used: Revaxis® (diphtheria, tetanus, polio) and Boostrix® (diphtheria, tetanus, pertussis) are both approved boosters but differ in their antigen composition. The choice between these products depends on the prescribing pediatrician and parental preference, such that some children received a booster covering polio but not pertussis, or vice versa (see Table S1). Coverage for pneumococcal vaccines, targeting *Streptococcus pneumoniae*, was completed in 57 patients (77%).

For varicella-zoster virus (VZV), 53 patients (72%) were considered immune based on a documented history of prior infection and/or vaccination; however, detailed information regarding the timing and specific source of immunity was unavailable.

Hepatitis B virus (HBV) vaccine coverage, often part of the primary infant series, was documented in 47 patients (64%).

**Fig. 2:**
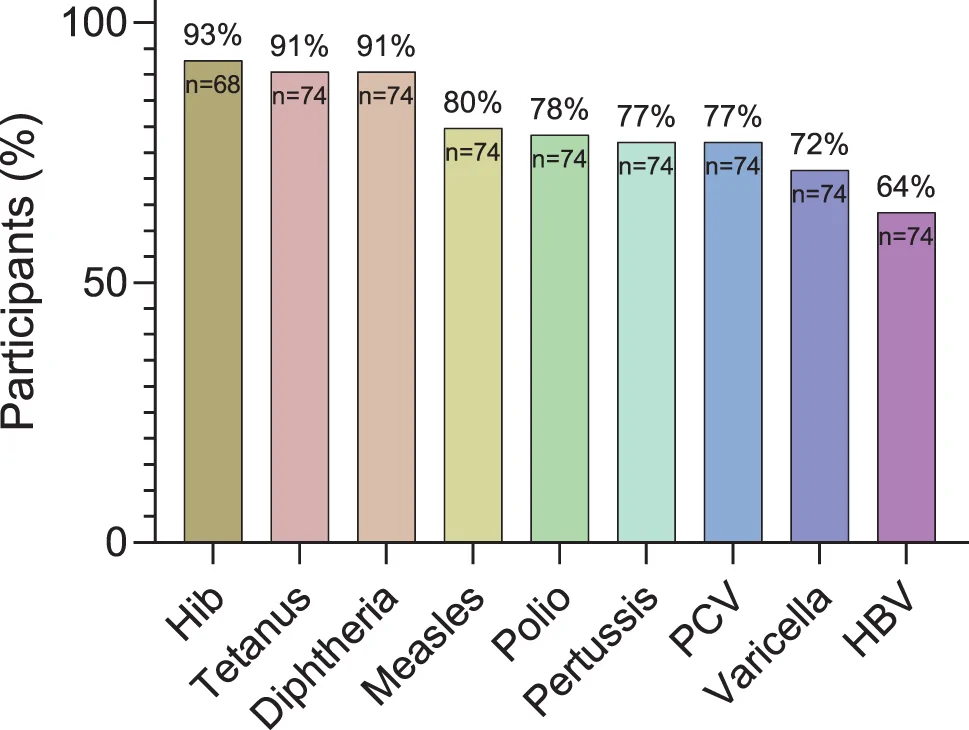
Percentage of patients “up to date” for their vaccine at time of diagnosis for each vaccine antigen

These detailed vaccine coverage rates are illustrated in Figure 2 and Table S1, providing an overview of immunization status across the recommended antigens for children with rheumatologic diseases at the time of diagnosis.

### Vaccine serology

**Fig. 3:**
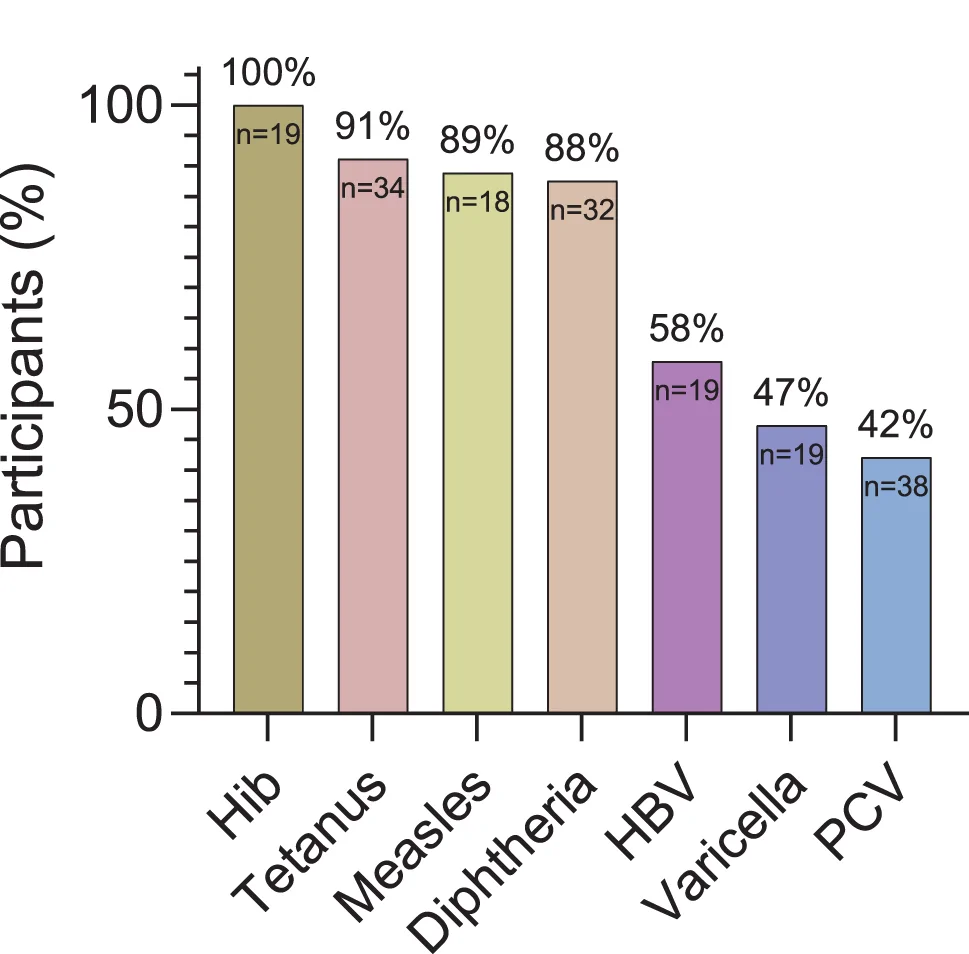
Percentage of seroprotected patients at diagnosis for each vaccine antigen. VZV immunity includes both vaccination and natural infection

At the time of diagnosis, serological data were available for a subset of the study population with 38 (51%) patients who had at least one serology. As shown in Fig. 3, seroprotection rates were highest for Hib (19/19 patients, 100%), followed by tetanus (31/34, 91%), measles (16/18, 89%), and diphtheria (28/32, 88%). Seroprotection rates were lower for hepatitis B (11/19, 58%), varicella (9/19, 47%), and pneumococcus (16/38, 42%). These findings indicate heterogeneity in baseline immunity, particularly for pneumococcus, varicella, and hepatitis B, despite prior vaccination in many cases.

As shown in Table 2, the number of patients with available serological follow-up decreased over time. Overall, the proportion of patients with protective antibody titers remained relatively stable during the 10 years following diagnosis across all vaccine antigens, although notable temporal fluctuations were observed. However, due to the retrospective design of the study, the exact timing of booster vaccinations could not be consistently determined, and the same children did not necessarily contribute serological samples at each time point. Consequently, it is challenging to accurately evaluate potential antibody waning over time, to assess individual responses to booster vaccinations, or to determine the duration of seroprotection following booster administration in relation to different immunosuppressive treatments.

**Table 2:** Percentage of patients seroprotected for the various vaccine antigens in years following diagnosis

Year	Hib N (%)	Tetanos N (%)	Measles N (%)	Diphteria N (%)	Hep B N (%)	Varicella N (%)	Pneumococcus N (%)
Basline	19/19 (100%)	31/34 (91%	16/18 (89%)	28/32 (88%)	11/19 (58%)	9/19 (47%)	16/38 (42%)
1	6/7 (86%)	19/22 (86%)	10/12 (83%)	21/22 (95%)	4/7 (57%)	6/14 (43%)	17/25 (68%)
2	9/10 (90%)	19/20 (95%)	6/7 (86%)	17/21 (81%)	3/5 (60%	6/12 (50%)	12/21 (57%)
3	7/7 (100%	18/21 (86%)	7/8 (88%)	16/21 (76%)	7/7 (100%)	9/10 (90%)	14/21 (67%)
4	5/5 (100%	11/14 (79%)	1/3 (33%)	9/13 (69%)	3/5 (60%)	2/3 (67%)	10/16 (63%)
5	5/5 (100%	16/16 (100%	3/5 (60%)	12/14 (86%)	2/4 (50%)	2/7 (29%)	11/19 (58%)
6	2/2 (100%)	8/8 (100%	0/1 (0%)	7/8 (88%)		1/1 (100%)	5/10 (50%)
7	3/3 (100%)	5/5 (100%	2/3 (67%)	5/5 (100%)	2/2 (100%)	3/4 (75%)	8/10 (80%)
8	2/2 (100%)	9/9 (100%)	1/1 (100%)	7/9 (78%)		1/2 (50%)	6/8 (75%)
9		5/5 (100%)		4/4 (100%)			5/5 (100%)
10	1/1 (100%)	5/5 (100%)	1/2 (50%)	5/6 (83%)	0/1 (0%)	2/2 (100%)	5/7 (71%)

Vaccine immunity was defined according to laboratory cut-offs used at the HUG and are as follows: diphtheria and tetanus: 100 U/L, measles and varicella: 150 U/L, Hib: 0.15 mg/L, Hepatitis B: 10 U/L. Pneumococci: ≥0.5 mg/L for >50% tested serotypes

## Discussion

This study provides a detailed assessment of vaccination coverage and serological follow-up in children with rheumatologic diseases, examining both the time of diagnosis—before the initiation of immunosuppressive therapy—and longitudinal trends during follow-up. Our findings highlight significant gaps in baseline vaccination coverage and vaccine-induced immunity, together with limited serological surveillance over time, underscoring the complexity of immunization management in this vulnerable population.

### Vaccination coverage at diagnosis

At diagnosis, before the initiation of immunosuppressive treatment, the overall vaccination coverage of our cohort was generally good; however, the coverage was suboptimal for varicella (72%), hepatitis B (64%), and to a lesser extent pneumococcus (77%) and measles (80. These findings are similar for pneumococcus but slightly lower for measles than those observed in other Swiss pediatric populations of healthy and non-immunosuppressed children [22–25]. However, previously published cohorts have evaluated vaccination status at diagnosis of rheumatologic diseases, and all reported lower-than-expected vaccination rates in children with juvenile idiopathic arthritis or other rheumatologic conditions [26–31]. Similar to our study, these cohorts assessed vaccination status prior to immunosuppressive therapy, emphasizing the need for early intervention to optimize immunity before treatment begins. The insufficient MMR and varicella vaccination rates in our cohort are also in agreement with prior work identifying MMR as one of the most frequently missed vaccines [26–28]. Multiple factors likely contribute to incomplete vaccination at diagnosis, including disease severity at presentation, competing clinical priorities, and concerns among parents, general pediatricians, and rheumatologists regarding potential disease exacerbation following vaccination. In some situations, diagnostic uncertainty may delay vaccination updates until immunosuppressive therapy is being considered—by which time the opportunity for timely immunization may already have been missed [7, 27, 29]. Additionally, incomplete documentation and missing vaccine records may lead to underestimation of true coverage, supported by our observation that some children with missing vaccine entries still demonstrated protective baseline serology. The time of diagnosis represents a critical immunization window in pediatric rheumatology, as it may constitute the last opportunity to safely administer live-attenuated vaccines before initiation of immunosuppressive therapy.

### Vaccine immunity at diagnosis

Serological evaluation at diagnosis provides critical insight into baseline immunity in pediatric rheumatology. In our cohort, high seroprotection was observed for Hib (100%), tetanus (91%), measles (89%), and diphtheria (88%), consistent with adequate primary vaccination in most children. However, seroprotection rates were substantially lower for hepatitis B (58%), varicella (47%), and pneumococcus (42%), despite prior vaccination or past infection in many cases. Evidence in the literature is limited, although one study reported reduced hepatitis B antibody levels in children with newly diagnosed JIA compared with healthy controls despite standard immunization^27^. These findings reinforce the value of systematic baseline serology at diagnosis. Given the elevated infection risk associated with immunosuppressive therapy and potential for suboptimal vaccine responses, baseline serology can guide catch-up vaccination and inform booster strategies. Integrating serological testing into routine pre-treatment evaluation aligns with growing evidence and current EULAR/PRES recommendations advocating for baseline serological screening [7, 10].

### Longitudinal vaccine serology follow-up

A key limitation of our dataset is that longitudinal serology was not systematically repeated in the same patients each year. As a result, although we were able to describe annual proportions of seroprotected children, it was not possible to determine persistence of protection within individuals, construct Kaplan–Meier curves, or robustly evaluate determinants of antibody waning such as immunosuppressive treatment type. Furthermore, precise dates of booster administration were difficult to retrieve, preventing reliable assessment of booster responses and durability. Future prospective studies are needed.

During follow-up, both the frequency of serological monitoring and the proportion of children with protective titers declined, highlighting insufficient long-term immunological surveillance despite increased vulnerability to vaccine-preventable infections. Seroprotection for diphtheria and tetanus remained largely stable, likely reflecting durable immunity induced by toxoid vaccines and regular booster schedules. This is consistent with previous findings, including a Russian cross-sectional study reporting variable seroprotection for hepatitis B, diphtheria, and measles and another work demonstrating preserved memory B-cell responses despite lower tetanus IgG in children who had not received recent boosters [15, 32]. Good post-vaccination responses to influenza in JIA patients on biologics have also been reported [33], suggesting preserved immunogenicity for some antigens despite immunosuppression.

However, some patients remained unprotected against measles throughout the entire follow-up period. This is concerning in light of recent measles resurgence [27, 28]. It is important to note that antibody levels are imperfect correlates of protection: cellular immunity—particularly memory T-cell responses—may persist and maintain protection despite waning or undetectable antibody titres. This applies especially to measles and varicella, for which T-cell-mediated immunity may confer protection even in the absence of seroprotective antibody levels. Indeed, some recent studies suggest preserved cellular immunity even under certain immunosuppressive therapies, including anti-CD20 [34]. However, the assessment of T-cell immunity is currently not standardized and lacks validated correlates of protection, precluding its routine clinical use in this setting. T-cell immunity assessment should therefore be considered as a complementary research tool in immunosuppressed children. Varicella seropositivity was stable over time. Pneumococcal seroprotection was low during the first years after diagnosis and increased over time. This finding is particularly concerning given increased risk of invasive pneumococcal disease in immunosuppressed children [3]. Our findings are consistent with prior studies reporting poor pneumococcal protection in various pediatric populations, both immunocompromised and heathy [35, 36]. This highlights the need for optimized strategies including booster doses and potentially higher-valent conjugate vaccines such as PCV15 or PCV20, particularly in high-risk patients [17]. While the Swiss pneumococcal vaccination schedule changed between 2005 and 2023, serological testing was adapted accordingly.

Collectively, these longitudinal findings illustrate marked heterogeneity in vaccine-induced immunity and reinforce the need for structured post-diagnosis immunological surveillance. While Hib, tetanus, and diphtheria protection appear sustained, measles, hepatitis B, varicella, and pneumococcus require closer monitoring, in order of decreasing seroprotection. Recent evidence and EULAR/PRES guidance support administration of booster doses of live-attenuated vaccines in selected patients with low-grade immunosuppression, challenging previously overly restrictive practices. Regular serological monitoring and timely boosters may help maintain protection and reduce infection risk.

### Implications and recommendations

These findings highlight the need for structured immunization strategies in pediatric rheumatology care. Baseline serology should form part of routine pre-treatment evaluation to identify incomplete protection and guide individualized catch-up schedules [7, 10]. Ongoing serological monitoring is also warranted, particularly for antigens associated with waning immunity. The limited follow-up surveillance in our cohort reflects broader challenges reported in other studies and likely reflects logistical barriers and lack of standardized protocols. In Switzerland, the absence of a unified national digital vaccine registry complicates communication between specialists and primary care; implementation of such systems could markedly improve vaccination continuity and oversight.

### Study limitations

This study has several limitations. Its retrospective design over a prolonged period limits completeness of documentation, particularly vaccination records, potentially underestimating coverage. Serological testing was not systematic, restricting longitudinal interpretation. Due to sample-size constraints, we were unable to stratify serological persistence according to type and intensity of immunosuppressive therapy, although these factors are known to influence vaccine responses. This was a single-center study, which may limit generalizability. Finally, infection outcomes were not assessed, preventing correlation between serostatus and clinical events. Additionally, for varicella, information regarding the origin of immunity (natural infection versus vaccination) and the age at infection was incomplete for a substantial proportion of patients, precluding reliable stratification of analyses according to this variable without introducing significant risk of bias. As highlighted by Bizjak et al., immunogenicity may differ between natural varicella infection and vaccination; this limitation should be considered when interpreting the varicella seroprotection data presented here [37]. Future prospective studies integrating clinical infectious data with structured systematic immunological follow-up are required to confirm the clinical impact of our findings.

## Conclusion

Children with rheumatologic diseases frequently begin immunosuppressive therapy without complete vaccination coverage, especially for varicella, hepatitis B, pneumococcus, and measles. Although baseline immunity to some antigens is preserved, long-term protection is inconsistently monitored and may decline over time. Improved vaccination documentation, systematic baseline serological testing, structured longitudinal surveillance, and timely booster administration are essential to ensure durable protection in this high-risk population. Integration of structured vaccination management into pediatric rheumatology care pathways may significantly reduce preventable infectious morbidity in this high-risk population.

## Electronic supplementary material

Below is the link to the electronic supplementary material.

Supplementary material 1

## Data Availability

The data will be made available on request.
