# Supplementary material for: Vaccination coverage and serological follow-up in children with rheumatologic diseases: a retrospective single-centre study
Source: Pediatr Rheumatol Online J. 2026 Jun 23;24:59. doi: 10.1186/s12969-026-01240-5 (PMC13551743; doi:10.1186/s12969-026-01240-5)
Supplement: Supplementary file 1 [file 12969_2026_1240_MOESM1_ESM.docx]

Suppl Table 1: Swiss vaccine recommendations for children with rheumatic diseases

| **Vaccine** | **Recommendation** | **Correlates of protection** |
| --- | --- | --- |
| **Varicella** | The up-to-date status for varicella is defined as either receiving the two recommended vaccine doses or having a documented history of natural infection. It is recommended to assess and administer vaccines before starting immunosuppressive therapy if the child is not already immune through a natural disease course. For live vaccines, decisions are made on a case-by-case basis once immunosuppression has started.  Note that for healthy children, the VZV vaccine is only recommended at 9 and 12 months of age since 2023. Before, it was only recommended for children >11 years who had not been exposed to the virus. | 150 IU/L |
| **Measles, Mumps, Rubella (MMR)** | Recommended before starting immunosuppressive therapy if the child is not immune. Live vaccines are given on a case-by-case basis if immunosuppression has already begun. For healthy children, the MMR vaccine is recommended with 2 doses at 9 and 12 months since 2019, before the first dose was recommended at 12 months and the second dose between 15-24 months. | 150 IU/L for Measles. Mumps and Rubella’s serological values are not measured. |
| **Diphtheria** | Routine vaccination as part of childhood immunization schedule. Booster doses as per national schedule. Vaccine DTPa-IPV-Hib+or-HBV at 2, 4 and 12 months since 2019. Before 2019, 3 doses in the first year of life (for example at 2, 4 and 6 months) plus a 4th dose during the second year of life. | 100 IU/L |
| **Tetanus** | Routine vaccination as part of childhood immunization schedule. Booster doses as per national schedule. Vaccine DTPa-IPV-Hib+or-HBV at 2, 4 and 12 months since 2019. Before 2019, 3 doses in the first year of life (for example at 2, 4 and 6 months) plus a 4th dose during the second year of life. | 100 IU/L |
| **Pertussis** | Routine vaccination as part of childhood immunization schedule. Booster doses as per national schedule. Vaccine DTPa-IPV-Hib+or-HBV at 2, 4 and 12 months since 2019. Before 2019, 3 doses in the first year of life (for example at 2, 4 and 6 months) plus a 4th dose during the second year of life. | Pertussis’ serological values are not measured. |
| ***Haemophilus influenzae type b (Hib)*** | Recommended in early childhood as part of routine vaccinations before 5 years of age. Vaccine DTPa-IPV-Hib+or-HBV at 2, 4 and 12 months since 2019. Before 2019, 3 doses in the first year of life (for example at 2, 4 and 6 months) plus a 4th dose during the second year of life. | 0.15 mg/L |
| **Polio** | Inactivated polio vaccine (IPV) is recommended according to routine schedule. Avoid live oral polio vaccine if on immunosuppressive therapy. Vaccine DTPa-IPV-Hib+or-HBV at 2, 4 and 12 months since 2019. Before 2019, 3 doses in the first year of life (for example at 2, 4 and 6 months) plus a 4th dose during the second year of life. Note that Revaxis® (diphtheria, tetanus, polio) provides polio booster coverage, while Boostrix® (diphtheria, tetanus, pertussis) does not; the choice between these products varies by prescriber and parental preference. | Polio’s serological values are not measured. |
| **Hepatitis B** | Strongly recommended, especially if immunosuppressive therapies are planned. Booster doses may be required depending on serology results. This vaccine is recommended as basic vaccination for children together with the DTP-IPV-Hib since 2019. Before, it was recommended as complementary vaccine for at risk groups at any age with 3 doses at 0, 1, 6 months (2 doses between 11-15 years). | 10 IU/L |
| ***S. pneumoniae*** | Pneumococcal vaccination is highly recommended due to increased risk of infection in patients on immunosuppressive therapy. May need revaccination or booster doses according to serology results.  The pneumococcal vaccine is recommended as complementary vaccine for all children <2 years since 2005, and to all children <5years since 2010. It is part of the basic vaccination for children <5 years since 2019. However, since 2000, pneumococcal vaccination for at risk children >5 years is recommended, first with the plain polysaccharide-23 valent vaccine, and since 2020 with the conjugate vaccine.  Note that the PCV7 is available since 2001, and the PCV13 since 2010, and the PCV15 since 2024. | Immunoglobulin G (IgG) levels >0.5 mg/L in response to at least 4 of the 7 tested serotypes (4, 6b, 9v,14, 18c, 19F, and 23F) are considered “seroprotected” |
| **Meningococcus** | Recommended for children under 5 and adolescents between 11 and 20, as part of the national immunization program. The meningococcal serogroup C vaccine (MenC) is recommended as basic vaccination since 2014 and as complementary vaccine already since 2006. The ACWY meningococcal vaccine (MenACWY) is recommended since 2019 as complementary vaccine and since 2024 as basic vaccine. The meningococcal B vaccine is recommended as complementary vaccine since 2024. | Meningococcus’ serological values are not measured. |
